# Supplementary material for: Content-rich biological network constructed by mining PubMed abstracts
Source: BMC Bioinformatics. 2004 Oct 8;5:147. doi: 10.1186/1471-2105-5-147 (PMC528731; doi:10.1186/1471-2105-5-147)
Supplement: Additional File 2 — The original results of the above study (non-essential files are deleted to keep the file size under the limit set by BMC bioinformatics). [file 1471-2105-5-147-S2.bz2 › chilibotAdditionalFile2/dip10/726ID9822594E18075/html/HLYD_HLYA.html]

 


 **HLYD** and **HLYA** 
  
Found 65 abstracts in PubMed, retrieved 10.  
 

 What does Google say? 
 PDF only 
| .edu only 

---

**Interactive relationship** (e.g. stimulation, inhibition, etc)

**Neutral relationship**- Our data suggest that TnhlyAs generated hemolysin  [ **HLYA** ]  fusion proteins are secreted efficiently by the HlyB  **HlyD**  TolC hemolysin  [ **HLYA** ]  secretion machinery.  Ref: 9711856 FEMS Microbiol Lett, 1998
- The system which consists of the two specific membrane proteins HlyB and  **HlyD**  and the outer membrane protein TolC, recognizes on  **HlyA**  a C terminally located signal sequence of about 60 amino acids.  Ref: 9382730 Behring Inst Mitt, 1997
- Our results are compatible with the idea that a TolC, together with the TolC homologous part of  **HlyD** , forms a pore in the outer membrane through which hemolysin  [ **HLYA** ]  is released and vancomycin taken up.  Ref: 9393456 Mol Gen Genet, 1997
- The export of Escherichia coli hemolysin  [ **HLYA** ]  across the cytoplasmic and the outer membranes requires the COOH terminal signal sequence of  **HlyA** , the two specific translocator proteins HlyB and  **HlyD** , and the outer membrane protein TolC.  Ref: 9630987 Nat Biotechnol, 1996
- We engineered plasmid pETR14, containing the hlyB and  **hlyD**  genes of the Escherichia coli hemolysin  [ **HLYA** ]  operon, to express a fusion protein containing 720 amino acid residues from the nontoxic, receptor binding, carboxy terminus of C. difficile toxin A and the secretion signal of E. coli hemolysin  [ **HLYA** ]  A.  Ref: 9199470 Infect Immun, 1997

**Non-interactive relationship** (e.g. studied together, co-existance, homology, etc.)

- The structure and function of the components of the  **HlyA**  secretion apparatus, HlyB,  **HlyD**  and TolC, have been studied in great detail.  Ref: 11755084 Trends Microbiol, 2002
- The toxin  **HlyA**  is exported from Escherichia coli, without a periplasmic intermediate, by a type I system comprising an energized inner membrane IM translocase of two proteins,  **HlyD**  and the traffic ATPase HlyB, and the outer membrane OM porin like TolC.  Ref: 9822594 EMBO J, 1998
- We show that an scFv  **HlyA**  hybrid secreted by Hly type I transporter TolC, HlyB,  **HlyD**  is accumulated in the extracellular medium with the disulphide bonds correctly formed.  Ref: 11309117 Mol Microbiol, 2001
- We have developed an export cloning system that is composed of two vectors one in which the fusion of the desired gene with the 3 end of  **hlyA**  is generated, and a second in which the sequences containing the fusion are combined with the accessory genes hlyB and  **hlyD** , thereby reconstructing the natural organization of the hly locus.  Ref: 9630987 Nat Biotechnol, 1996
